# Development of In Vitro and In Vivo Evaluation Systems for Vitamin D Derivatives and Their Application to Drug Discovery

**DOI:** 10.3390/ijms222111839

**Published:** 2021-10-31

**Authors:** Kaori Yasuda, Miyu Nishikawa, Hiroki Mano, Masashi Takano, Atsushi Kittaka, Shinichi Ikushiro, Toshiyuki Sakaki

**Affiliations:** 1 Department of Pharmaceutical Engineering, Faculty of Engineering, Toyama Prefectural University, 5180 Kurokawa, Imizu 939-0398, Toyama, Japan; kyasuda@pu-toyama.ac.jp (K.Y.); z16003@st.pu-toyama.ac.jp (H.M.); 2 Department of Biotechnology, Faculty of Engineering, Toyama Prefectural University, 5180 Kurokawa, Imizu 939-0398, Toyama, Japan; m-nishikawa@pu-toyama.ac.jp (M.N.); ikushiro@pu-toyama.ac.jp (S.I.); 3 Faculty of Pharmaceutical Sciences, Teikyo University, Tokyo 173-8605, Japan; mtakano@pharm.teikyo-u.ac.jp (M.T.); akittaka@pharm.teikyo-u.ac.jp (A.K.)

**Keywords:** vitamin D, vitamin D receptor, split luciferase-based biosensor, CYP24A1-dependent metabolism, CYP27B1, rickets, genome editing

## Abstract

We have developed an in vitro system to easily examine the affinity for vitamin D receptor (VDR) and CYP24A1-mediated metabolism as two methods of assessing vitamin D derivatives. Vitamin D derivatives with high VDR affinity and resistance to CYP24A1-mediated metabolism could be good therapeutic agents. This system can effectively select vitamin D derivatives with these useful properties. We have also developed an in vivo system including a *Cyp27b1-*gene-deficient rat (a type I rickets model), a *Vdr*-gene-deficient rat (a type II rickets model), and a rat with a mutant *Vdr* (R270L) (another type II rickets model) using a genome editing method. For *Cyp27b1*-gene-deficient and *Vdr* mutant (R270L) rats, amelioration of rickets symptoms can be used as an index of the efficacy of vitamin D derivatives. *Vdr-*gene-deficient rats can be used to assess the activities of vitamin D derivatives specialized for actions not mediated by VDR. One of our original vitamin D derivatives, which displays high affinity VDR binding and resistance to CYP24A1-dependent metabolism, has shown good therapeutic effects in *Vdr* (R270L) rats, although further analysis is needed.

## 1. Introduction

The active form of vitamin D_3_ (1α,25(OH)_2_D_3_) plays essential roles in calcium and phosphate homeostasis, cellular proliferation and differentiation, and immune responses. Since it could cause hypercalcemia and hypercalciuria, its clinical utility is limited [1,2]. A huge number of vitamin D derivatives have been synthesized. Many of them have been studied in clinical trials for the treatment of type I rickets, osteoporosis, psoriasis, renal osteodystrophy, and also leukemia, pancreatic, prostate, and breast cancers [3,4,5,6,7,8,9]. A number of vitamin D derivatives have been approved by the FDA for clinical use in a variety of disorders, for example, 22-oxacalcitriol (Maxacalcitol) and calcipotriol (Dovonex) for treatment of psoriasis, 19-nor-1α,25(OH)_2_D_2_ (Zemplar), 26,26,26,27,27,27-hexafluoro-1α,25(OH)_2_D_3_ (Falecalcitriol), and doxercalciferol (Hectorol) for secondary hyperparathyroidism, and 1α(OH)D_3_ (alfacalcidol) and eldecalcitol (Edirol) for osteoporosis. Although many vitamin D derivatives have antiproliferative activity, none have been approved for cancer treatment. So far, only a small number of clinical studies have taken place, such as EB1089 in a phase II study for pancreatic cancer [6,10], and Hectorol and Zemplar in phase I/II advanced androgen-insensitive prostate cancer trials [7,11,12]. Unfortunately, neither have produced any significant objective responses. However, a new 1α,25(OH)_2_D_3_ analog, 19-nor-14-epi-23-yne-1α,25(OH)_2_D_3_ (inecalcitol), is being developed for prostate cancers and chronic leukemia [13,14].

The active form of vitamin D_3_ (1α,25(OH)_2_D_3_) plays essential roles in calcium and phosphate homeostasis, cellular proliferation and differentiation, and immune responses. Its clinical utility is limited because it can cause hypercalcemia and hypercalciuria. [1,2]. Several thousand vitamin D derivatives have been synthesized, and many have been studied in clinical trials to treat conditions, including type I rickets, osteoporosis, leukemia, psoriasis, renal osteodystrophy, and pancreatic, prostate, and breast cancers. [3,4,5,6,7,8,9]. A number of vitamin D derivatives have been approved by the FDA for clinical use in a variety of disorders. These derivatives include calcipotriol (Dovonex; Leo Pharmaceuticals) and 22-oxacalcitriol (Maxacalcitol; Chugai Pharmaceuticals) for treatment of psoriasis; 19-nor-1α,25(OH)_2_D_2_ (Zemplar; Abbot Laboratories; Chicago, IL, USA), 26,26,26,27,27,27-hexafluoro- (Falecalcitriol; Sumitomo Pharmaceuticals and Taisho Pharmaceuticals), and doxercalciferol (Hectorol; Bone Care Int.; Middleton, WI, USA) for secondary hyperparathyroidism; and 1α(OH)D_3_ (alfacalcidol; Chugai Pharmaceuticals Co., Ltd.; Tokyo, Japan) and eldecalcitol (Chugai Pharmaceuticals Co., Ltd.; Tokyo, Japan) for osteoporosis. Although many vitamin D derivatives, including those approved by the FDA for treating secondary hyperparathyroidism and renal osteodystrophy, have displayed antiproliferative activity, none have been approved for cancer treatment. To date, only a limited number of clinical studies have taken place, including a phase II study of EB1089 in pancreatic cancer. [6,10]. Hectorol and Zemplar have been studied in phase I/II advanced androgen-insensitive prostate cancer trials [7,11,12]. Unfortunately, neither produced any significant objective responses. Recently, a new 1α,25(OH)_2_D_3_ analog, inecalcitol, is being developed for prostate cancers and chronic leukemia [13,14].

In evaluating these vitamin D derivatives, (1) affinity for vitamin D receptor, (2) affinity for vitamin-D-binding protein (DBP), (3) resistance to metabolism by CYP24A1, and (4) ability to differentiate leukemia-derived HL-60 cells into macrophages are considered to be essential properties. In addition, they must show therapeutic efficacy in animal studies. In the case of derivatives under development for cancer treatment, therapeutic efficacy will be evaluated using tumor-bearing animals. Construction of appropriate evaluation models is indispensable for developing vitamin D derivatives for pharmaceutical use. We have developed in vitro systems that can easily measure vitamin D receptor (VDR) affinity [15,16,17,18,19] and CYP24A1-mediated metabolism [20,21,22,23,24]. We have also generated genetically modified rats using genome editing as follows: *Cyp27b1-*gene-deficient rats (a type 1 rickets model animal), vitamin D receptor-gene-deficient rats, and rats harboring a mutant vitamin D receptor (R270L) gene (type II rickets model animals) [25]. We have also generated *Cyp24a1*-gene-deficient rats to elucidate enzymes and metabolic pathways responsible for vitamin D derivative metabolism [26]. In this review, we describe the in vitro and in vivo systems we have developed for evaluation of vitamin D derivatives, and discuss the derivatives we have synthesized to date.

## 2. In Vitro System to Easily Examine the Affinity for VDR of Vitamin D Derivatives

### 2.1. Measurement of Binding Affinity of Vitamin D Derivatives for VDR

The widely used method for evaluating the binding ability of vitamin D derivatives for VDR in a cell-based assay system is a reporter assay that induces expression of luciferase (Luc) under the control of a promoter containing a vitamin D response element (VDRE) [27,28,29]. It is noted that it takes more than 12 h for the reporter protein to be expressed, and the direct binding between the receptor and the ligand cannot be evaluated. Although a competitive system using native VDR and tritium-labeled 1α,25(OH)_2_D_3_ was widely used, it is no longer commercially available. Thus, we tried to develop a new detection system that easily evaluates the affinity of vitamin D derivatives for VDR in a short time. We focused on the split-type luciferase technology [15,16,17,18,19,30,31,32,33,34,35,36,37]. This system can evaluate the affinity of the ligand by increasing or decreasing the luminescence of the split-type luciferase.

### 2.2. Development of a Novel Bioluminescent Sensor to Detect and Discriminate between Vitamin D Receptor Agonists and Antagonists in Living Cells (1st Generation)

**Figure 1:**
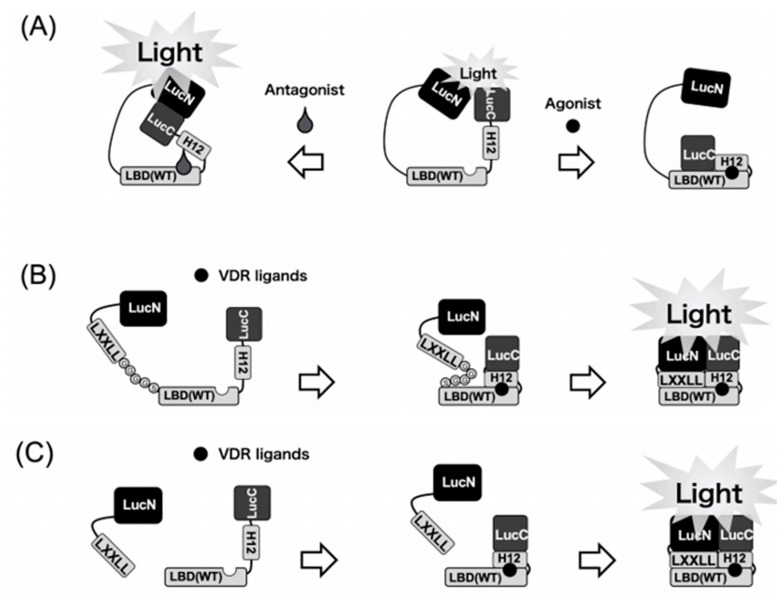
Schematic diagrams of the biosensors to detect VDR ligands. (**A**) 1st generation. Binding of the VDR agonists to the LBD may cause a conformational change of the LBD that leads to disruption of the functional complex between N-terminal and C-terminal domains of the luciferase. In contrast, binding of the antagonist leads to the reassembly of N-terminal and C-terminal domains of the luciferase to increase the activity. (**B**) 2nd generation. Binding of VDR ligands to the biosensor may cause a conformational change of helix12 (H12) in LBD. After conformational change of LBD, the LXXLL motif interacts with LBD in the biosensor. Then, this intramolecular dynamic change of the WT biosensor leads to reconstitution of the functional complex between LucN and LucC fragments of the split luciferase. (**C**) 3rd generation. Binding of the VDR ligands to the LBD–LucC may cause a positional change of helix12 in LBD. Then, the LucN–LXXLL and LBD–LucC forms a functional complex to exhibit the luciferase activity.

Two chimeric fusion proteins that contained both split-luciferase and the ligand binding domain (LBD) of the VDR were constructed. This fusion protein was labeled as LucN–LBD–LucC. It contained the N-terminal domain taken from luciferase (LucN), LBD, and C-terminal domain from luciferase (LucC) from N-terminus to C-terminus. LucC–LBD–LucN has the C-terminal domain of luciferase at the N-terminus of the fusion protein (Figure 1) [15]. Unexpectedly, the LucC–LBD–LucN worked better than LucN–LBD–LucC. Luciferase activity was significantly diminished by the addition of the VDR agonists to COS-7 cells that expressed LucC–LBD–LucN. On the other hand, the VDR antagonist notably enhanced the activity of the chimeric luciferase in a dose- and time-dependent manner. Our novel model for detecting and discriminating between VDR agonists and antagonists is very useful for testing synthetic analogs of vitamin D that show reasonable affinity for normal or mutant VDRs.

Patients with type II rickets showing the R274L mutation caused a 1000-fold reduction in the binding activity for 1α,25(OH)_2_D_3_ and remarkably lowered vitamin-D-related gene expression [38]. It is Arg274, located in LBD of VDR, that is responsible for attaching 1α,25(OH)_2_D_3_. This happens by a formation of an additional hydrogen bond with 1α-hydroxyl of 1α,25(OH)_2_D_3_. LucC–LBD (R274L)–LucN was constructed to investigate vitamin D ligands of high affinity for the mutant VDR (R274L). A total of 5 out of the 33 vitamin D analogs tested showed much higher binding for the mutant VDR (R274L) than the vitamin D hormone. The highest binding activity was shown by 2α-(2-(tetrazol-2-yl)ethyl)-(AH-1). These analogs might be considered as future drug candidates against HVDRR that is caused by the mutant VDR (R274L) [16].

### 2.3. Development of a Highly Sensitive In Vitro System to Detect and Discriminate between Vitamin D Receptor Agonists and Antagonists

We have established an in vitro screening system for VDR ligands using the LucC–LBD–LucN proteins expressed in *Escherichia coli (E. coli)* cells [17]. It should be noted that this system could be completed within 30 min, and its activity was unchanged after 10 freeze–thaw cycles. This highly sensitive and convenient system would be quite useful to screen VDR ligands with therapeutic potential for osteoporosis, renal osteodystrophy, cancers, and immune disorders.

### 2.4. Design of a Biosensor Based on Split Luciferase for Detection of VDR Ligands (2nd Generation)

The model we developed is very useful for a fast investigation of VDR ligands. However, the sensitivity of our biosensor (LucC–LBD–LucN) is not as high as expected. LBD is known to interact via the LXXLL motif with transcription coactivators, such as SRC-1, TIF-2, or DRIP-205 to initiate vitamin-D-related gene expression, when binding natural VDR ligands. This is why we anticipated that it is the LXXLL motif that changes the enzymatic profile of luciferase–LBD biosensors. This is why LucN–LBD–LucC and not LucC–LBD–LucN was used as a basing fragment. We created a new biosensor consisting of the LBD (121–427 aa) of VDR, N- and C-terminal of firefly luciferase fragments (LucN (1–415 aa) and LucC (416-550 aa)), the LXXLL peptide sequence, and peptide sequence (Gly-Gly-Gly-Gly-Ser (GGGGS)) × 3 as the flexible linker [18]. This construct we labeled as LucN–LXXLL–(GGGGS) × 3–LBD–LucC WT biosensor and WT means the wild-type of LBD (Figure 1). Light intensity of luciferase is low when natural VDR ligands are absent. The luciferase light intensity is immediately and remarkably increased when the ligand is bound to the WT biosensor. To sum up, we have successfully created a very sensitive biosensor which shows the increase in light intensity when binding VDR agonists.

To this end, we developed a novel and WT biosensor of high sensitivity by examining three types of LXXLL peptides (NHPMLMNLLKDN, LTEMHPILTSLLQNGVDHV, and LSETHPLLWTLLSSTEGDSM) that interact with the LBD in response to 1α,25(OH)_2_D_3_ or synthetic VDR agonists. The COS-7 cells that expressed each type of biosensor were treated with 1α,25(OH)_2_D_3_ (100 nM) and then the luminescence was measured 90 min later. Among the 10 biosensors we constructed, one showed a reduction in intensity of light in response to 1α,25(OH)_2_D_3_. Seven biosensors showed an excellent increase in light intensity. Our best biosensor showed the light intensity ca. one-third of that of full-length native luciferase of firefly. Quite unexpectedly, 25(OH)D_3_, as the low-affinity VDR ligand, also enhanced the intensity of light in a concentration-dependent manner. The half maximal relative intensity of light was recorded at 1 nM of 1α,25(OH)_2_D_3_ and at 20 nM of 25(OH)D_3_, respectively. We then compared the binding activity of 1α,25(OH)_2_D_3_ and 25(OH)D_3_ for the mutant VDR (R274L). As previously mentioned, the substitution of Arg274 to Leu causes a 1000-fold decrease in affinity of 1α,25(OH)_2_D_3_. As expected, in the R274L biosensor the concentration–response curve of 1α,25(OH)_2_D_3_ was very similar to that of 25(OH)D_3_. Thus, the biosensor system we developed may be very useful in elucidating novel vitamin D analogs as drug candidates against type II rickets resulting from VDR mutation, such as R274L.

### 2.5. Development of a Novel Two-Molecule System with a Highly Sensitive Biosensor (3rd Generation)

In the next step, we developed a two-molecule system named LXXLL + LBD biosensor, as shown in Figure 1, with a combination of two components [19]. The two plasmids were co-transfected and two proteins were co-expressed in COS-7 cells. The LXXLL + LBD biosensor-expressing COS-7 cells were treated with 100 nM of 1α,25(OH)_2_D_3_, and luciferase light intensity was measured at 90 min after treatment. Among all combinations of LXXLL + LBD biosensor, relative light intensity of A1 + B1 [19] was the highest in all combinations. The relative light intensity of combination A1 + B1 was approximately a 90- to 100-fold increase in response to 100 nM of 1α,25(OH)_2_D_3_. It should be noted that the detection limit was 0.005 nM (5 pM) of 1α,25(OH)_2_D_3_, indicating that the sensitivity of LXXLL + LBD biosensor is higher than that of our previous biosensors. [15,16,17,18]. Our LXXLL + LBD biosensor might be used for the measurement of 1α,25(OH)_2_D_3_ and 25(OH)D_3_ in the plasma.

## 3. In Vitro Evaluation of CYP24A1-mediated Metabolism of Vitamin D Derivatives

### 3.1. Expression of Rat or Human CYP24A1 in E. coli Cells

The rat *Cyp24a1* cDNA was cloned from the rat kidney cDNA library [39], and the isolated cDNA clone contained the open reading frame consisting of 514 amino acids. Since the amino acid sequence showed less than 40 % homology with already known CYPs, the new CYP family name, CYP24, was given to this vitamin-D-24-hydroxylase.

The molecular mechanism of *CYP24A1* gene regulation is quite complicated, and many factors are tissue-specifically involved in the expression of *CYP24A1* [40,41,42,43,44]. These facts strongly suggest that CYP24A1 is a physiologically essential enzyme that regulates the level of the active form of vitamin D.

When the deduced amino acid sequence from its cDNA was compared to that amino-terminal amino acid sequence of the CYP24A1 purified from rat kidney, it was found that the mature form of rat CYP24A1 lacks amino-terminal 32 amino acids. These results suggest that amino-terminal 32 amino acids function as a mitochondrial targeting signal, which is removed after translocation of CYP24A1 to mitochondria. We have successfully expressed the mature forms of rat and human CYP24A1 in *E. coli* cells to reveal their enzymatic properties [20,21,22,23,24].

### 3.2. Construction of a CYP24A1 Enzyme System Containing Adrenodoxin (ADX) and NADPH-Adrenodoxin Reductase (ADR)

**Figure 2:**
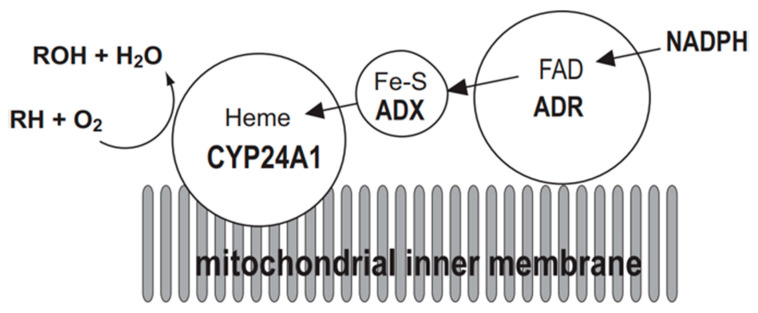
Mitochondrial electron transport chain of CYP24A1. CYP24A1-dependent mono-oxygenase activity requires the electron transfer from NADPH via NADPH-adrenodoxin oxidoreductase (ADR) and adrenodoxin (ADX) to the heme iron of CYP24A1 situated on the inner membrane of mitochondria. RH represents substrate of CYP24A1.

The mitochondrial P450 system consists of three components: CYP, ADX, and ADR. Electrons are sequentially transferred from NADPH through ADR and ADX to CYP24A1 (Figure 2). Thus, CYP24A1-dependent activity was measured in an in vitro reconstituted system containing purified ADX and ADR proteins. On the other hand, in a whole-cell system, co-expression of mature forms of CYP24A1, ADX, and ADR in *E. coli* is required. We have demonstrated that the *E. coli* expression system is quite useful to investigate enzymatic properties of CYP24A1. Using this *E. coli* expression system, we have determined kinetic parameters of CYP24A1 in the metabolism of the native vitamin D and various vitamin D derivatives, and revealed their metabolic pathways [45,46,47,48,49,50,51,52,53,54,55,56,57,58].

### 3.3. CYP24A1-Dependent Multi-Step Reaction toward the Active form of Vitamin D_3_

**Figure 3:**
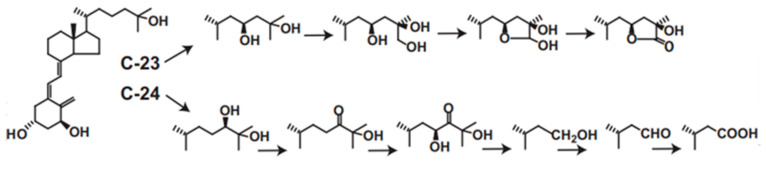
C-23 and C-24 oxidative pathways of 1α,25(OH)_2_D_3_ catalyzed by human CYP24A1. Human CYP24A1 catalyzes 6-step mono-oxygenation from C-24 hydroxylation to produce calcitroic acid, and 4-step mono-oxygenation from C-23 hydroxylation to the lactone formation.

CYP24A1 plays central roles in vitamin D metabolism and produces a wide variety of metabolites. We revealed that rat or human CYP24A1 catalyzes a six-step reaction, starting with hydroxylation at the 24*R* position of 1α,25(OH)_2_D_3_ to produce the final metabolite, calcitroic acid (Figure 3). In addition, human CYP24A1 catalyzes a four-step reaction, starting with hydroxylation at the 23*S* position to produce the 26,23-lactone form (Figure 3). In the reaction of P450, it is often seen that the reaction product is not released from the substrate binding pocket and the reaction proceeds further. Thus, the two- or three-step reaction is not special in the P450 reaction; however, there is no other P450 that catalyzes such a multi-step reaction for one substrate. Moreover, it is noted that the reaction by human CYP24A1 proceeds in a dual pathway, the C-24 pathway and the C-23 pathway. Interestingly, the ratio of the C-24 to C-23 pathways varies among animal species. In human CYP24A1, it is about 4:1, but, in rat CYP24A1, about 25:1; however, in animal species such as guinea pig and opossum, the C-23 pathway is major. In rat and human CYP24A1, the 326th amino acid residue from the N-terminus is Ala, whereas it is Gly in guinea pigs and opossum, and, when the Ala326 in rat and human CYP24A1 is replaced by Gly, it changes to the guinea pig type [59]. Given that inactivating the active form of vitamin D is the physiological role of CYP24A1, it may be less important whether the C-24 or C-23 pathway is predominant.

### 3.4. Metabolism of Vitamin D Derivatives by CYP24A1

**Figure 4:**
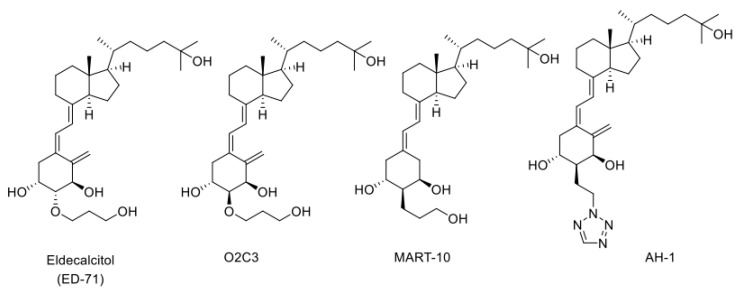
Structures of three CYP24A1-resistant VDR agonists: Eldecalcitol (ED-71), O2C3, MART-10, and AH-1.

The CYP24A1 gene has two VDREs in the promoter region [40,41,60] and, when the active form of vitamin D binds to VDR, remarkable transcriptional induction of CYP24A1 occurs. When a large amount of CYP24A1 protein is expressed in the cell, the active form of vitamin D is inactivated via the metabolic pathways described above. This mechanism appears to be crucial for keeping the level of the active form of vitamin D. However, when a vitamin D derivative with a high affinity for VDR is developed as a drug, the drug binds to VDR to induce CYP24A1. Therefore, vitamin D derivatives that are not easily metabolized by CYP24A1 could be excellent drugs with long-lasting efficacy. Eldecalcitol, an osteoporosis treatment drug developed by Chugai Pharmaceutical Co., Ltd., has a 3-hydroxy-propyloxy group at the 2β position of 1α,25(OH)_2_D_3_ (Figure 4). We revealed that CYP24A1 hardly metabolizes Eldecalcitol [53] and suggest that the resistance to CYP24A1-dependent metabolism may be a key factor that keeps its efficacy for a long time [53,61,62,63]. We have investigated the metabolism of many vitamin D derivatives by CYP24A1 and have clearly demonstrated the importance of CYP24A1-dependent metabolism. In addition, as mentioned above, the fact that there are animal species differences in the metabolic mode of 1α,25(OH)_2_D_3_ by CYP24A1 suggests that there are also animal species differences in the metabolism of vitamin D derivatives. Therefore, the development of vitamin D derivatives requires not only animal studies, but also metabolic studies using human CYP24A1 enzyme.

### 3.5. CYP24A1-Resistant Vitamin D Derivatives with a Substituent at C2α Position

We have synthesized many of A-ring-modified derivatives with a substituent at the C2α position, which have unique biological activities [64,65,66,67,68]. Of these derivatives, 2α-(3-hydroxypropoxy)-1α,25(OH)_2_D_3_ (O2C3), which is a C2-epimer of Eldecalcitol, was examined for the metabolism by CYP24A1. Five metabolites were detected in its metabolism by human CYP24A1, including both C-23 and C-24 oxidation pathways [48]. The *K*_m_ and *k*_cat_ values of human CYP24A1 for O2C3 were estimated to be approximately 16 times greater and 3 times lower than those for 1α,25(OH)_2_D_3_, respectively [48]. Accordingly, the catalytic efficiency (*k*_cat_/*K*_m_) of human CYP24A1 for O2C3 is only about 3% of 1α,25(OH)_2_D_3_. These results strongly suggest that O2C3 is much more resistant to CYP24A1-dependent metabolism than 1α,25(OH)_2_D_3_. It is noted that another C-2-substituted derivative, 19-nor-2α-(3-hydroxypropyl)-1α,25(OH)_2_D_3_ (MART-10) (Figure 4), was more resistant to CYP24A1-dependent degradation than O2C3 [69,70,71,72,73,74]. The *k*_cat_/*K*_m_ values of human CYP24A1 for MART-10 were about 0.3 % of those for 1α,25(OH)_2_D_3_.

Our in vivo studies using rats revealed that MART-10 had a potent anticancer effect, with a low calcemic effect, which is a suitable property as an anticancer drug. The resistance to CYP24A1 is also a suitable property of MART-10 as an anticancer drug.

## 4. In Vivo Evaluation System for Vitamin D Derivatives Using Genetically Modified Rats Generated by Genome Editing

### 4.1. Appearance and Growth of Genetically Modified (GM) Rats

**Figure 5:**
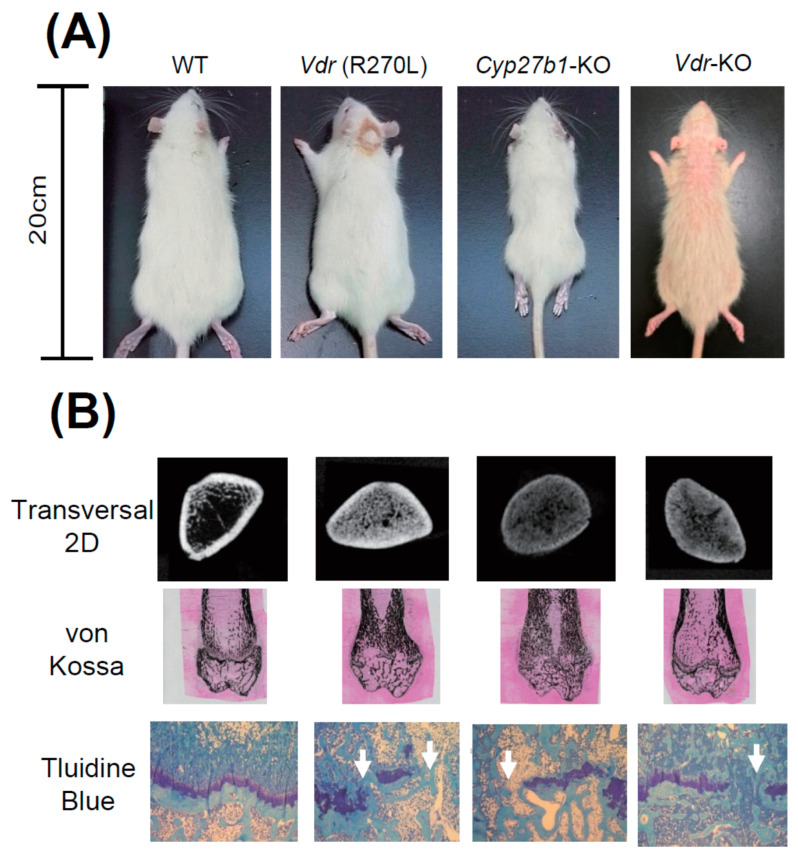
The appearance of GM rats and their abnormal bone formation [25]. (A) Comparison of body size and skin phenotype at 15 weeks of age. (B) First panels, 2D μ-CT images of horizontal section at distal femur; second panels, von Kossa staining of distal femur; bottom panels, toluidine blue staining of epiphyseal cartilage.

Figure 5A shows WT, *Vdr* (R270L), and *Vdr-*KO rats fed an F-2 diet containing 0.75% Ca, and *Cyp27b1*-KO rats fed a diet containing 1.15% Ca at 15 weeks after birth. Although *Cyp27b1-*KO rats were much smaller than WT rats, body sizes of *Vdr* (R270L) and *Vdr-*KO rats were not so different from that of the WT rats. Figure 5A shows the *Vdr-* abnormal skin and alopecia of KO rats. Elasticity and softness of the skin of *Vdr*-KO rats were substantially lowered and the wavy skin was formed [25]. Keratinization was elevated and follicles decreased, and formation of cysts appeared in the dorsal skin of *Vdr-*KO rats [25].

Figure 5A shows that growth was substantially diminished in *Cyp27b1*-KO rats compared to WT rats. However, only a slight decrease was observed in *Vdr* (R270L) and *Vdr-*KO rats. It was noted that approximately a half of male *Cyp27b1*-KO rats fed with the diet containing 0.75 % Ca died prior to 9 weeks of age, and none survived to 10 weeks of age (data not shown), whereas no animals had died at 15 weeks of age in the *Cyp27b1*-KO rats fed with the diet containing 1.15% Ca. Thus, the diet that contained 0.75% Ca was used for mutant *Vdr* (R270L) and *Vdr-*KO rats, while the diet containing 1.15 % Ca was used for *Cyp27b1*-KO rats. 

### 4.2. Osteogenesis and Plasma Ca, PTH, and 1α,25(OH)_2_D_3_ Levels in the GM Rats

It is noted that *Cyp27b1*-KO rats are remarkably smaller than other rats. Figure 5B shows the middle region of the femur in 2D μCT scan images. The femur lengths of *Cyp27b1-*KO, *Vdr* (R270L), and *Vdr-*KO rats were found to be remarkably shorter than those of WT rats. The μCT scanning and von Kossa staining of femurs showed hyperplasia of calcified trabecular bones with a narrow medullary cavity in all the *Vdr* (R270L), *Cyp27b1*-KO, and *Vdr-*KO rats (Figure 5B). The *Vdr* (R270L) and *Vdr-*KO rats expressed no clear differences in total bone mineral density (BMD). In contrast, the BMD of cortical bone in *Cyp27b1*-KO rats was substantially diminished [25].

Histological analysis of the epiphyseal cartilage demonstrated structural disorder of the growth plate in all the *Vdr* (R270L), *Cyp27b1*-KO, and *Vdr-*KO rats. Whereas WT growth plates contained aligned cartilage cells in the layered structure, growth plates in all three GM rats lost the sequential plate structure and cartilage cell alignment (Figure 5). Thus, the morphology of bone was abnormal in all three GM rats, and, in *Cyp27b1*-KO rats, the most significant disorders of bone were observed.

It is well known that rickets type I model *Cyp27b1-*KO mice, and rickets type II model *Vdr-*KO mice, have significantly lower plasma Ca levels than WT mice [75,76]. Expectedly, the plasma Ca level was substantially reduced, and the level of parathyroid hormone (PTH) in plasma was greatly increased in *Vdr* (R270L) rats and *Cyp27b1*-KO rats [25]. Unexpectedly, the plasma Ca level in *Vdr*-KO rats was normal at 15 weeks. In *Vdr*-KO rats, until 10 weeks, the plasma level of Ca was significantly lower than that in WT rats, and PTH level was substantially higher than that in WT rats [25]. Plasma PTH level in *Vdr*-KO rats was remarkably higher than that in WT rats; although, at 15 weeks, the level of Ca in plasma in *Vdr*-KO rats returns to normal. These findings might indicate that hyperparathyroidism occurred in *Vdr*-KO rats [25]. In addition, the putative incomplete formation of intercellular barriers in epithelial tissues, including the small intestine, in VDR-KO rats might cause the increased calcium permeability to result in the normal level of plasma Ca concentration [77].

Although plasma 1α,25(OH)_2_D_3_ level was significantly increased in *Vdr* (R270L) and *Vdr-*KO rats, it was significantly decreased in *Cyp27b1*-KO rats (8.0 ± 3.2 pg/mL (mean ± SEM, n = 7)) compared to WT rats (24.8 ± 5.2 pg/mL, (mean ± SEM, n = 7)) [25].

### 4.3. Effects of 25(OH)D_3_ Administration on Cyp27b1- KO Rats

As described previously [76], dietary administration of 25(OH)D_3_ recovered growth failure, skeletal disorders, and hypocalcemia of *Cyp27b1*-KO mice. Dietary administration of 25(OH)D_3_ to *Cyp27b1*-KO rats at 200 μg·kg^−1^·day^−1^ also significantly reversed growth failure [25]. The 25(OH)D_3_ administration normalized BMD of the cortex and trabecular bone of *Cyp27b1*-KO rats. Histological analysis of the femur clearly indicated a normal structure of the cortex and trabecular bone in *Cyp27b1*-KO rats [25]. The growth plate and chondrocytes were also normalized, and the plasma Ca and PTH levels of *Cyp27b1*-KO rats were fully normalized after 25(OH)D_3_ administration [25].

Plasma 1α,25(OH)_2_D_3_ level in *Cyp27b1*-KO rats was normalized by 25(OH)D_3_ administration. The 1α-hydroxylation activity toward 25(OH)D_3_ was observed in the liver mitochondrial fraction prepared from *Cyp27b1*-KO rats. It is noted that these results were similar to those obtained in our previous study using *Cyp27b1-*KO mice [76]. Because hepatic Cyp27a1 has a weak 1α-hydroxylation activity toward 25(OH)D3, Cyp27a1 is the most probable candidate to produce 1α,25(OH)_2_D_3_ from 25(OH)D3 in *Cyp27b1*-KO rats.

It is noted that 25(OH)D_3_ administration is highly effective in type I rickets model mice and rats. Because human CYP27A1 can convert 25(OH)D_3_ into 1α,25(OH)_2_D_3,_ similar effects might be expected in humans.

### 4.4. Effects of 25(OH)D_3_ Administration on Vdr (R270L) Rats

The 25(OH)D_3_ administration also normalized bone disorders with increased cortical BMD of *Vdr* (R270L) rats [25]. The reduced plasma Ca level in *Vdr* (R270L) rats was normalized by 25(OH)D_3_ diet, and the elevated plasma PTH and 1α,25(OH)_2_D_3_ levels observed before 25(OH)D_3_ administration were reduced to the normal levels.

The plasma concentration of 25(OH)D_3_ in *Vdr* (R270L) rats fed a 25(OH)D_3_-containing diet was about 500 nM. This concentration was 20 times higher than that in WT rats. It is noted that the affinity of 1α,25(OH)_2_D_3_ for *Vdr* (R270L) is nearly the same as that of 25(OH)D_3._ Thus, 25(OH)D_3_ is thought to be a leading ligand of *Vdr* (R270L) in these rats, because plasma 1α,25(OH)_2_D_3_ level in the *Vdr* (R270L) rats after the 25(OH)D_3_ treatment was much lower than that of 25(OH)D_3_ [25]. The remarkably higher levels of 24,25(OH)_2_D_3_ and 24-oxo-25(OH)D_3_ were consistent with the induction of *Cyp24a1* expression, which indicates the “*Vdr* (R270L)-dependent effects of 25(OH)D_3_”. The remarkable effects of 25(OH)D_3_ administration on rickets symptoms in *Vdr* (R270L) rats indicate that 25(OH)D_3_ might be efficacious in the treatment of patients with type II rickets caused by the human VDR mutant (R274L).

### 4.5. Predicted Effects of the Vitamin D Derivative AH-1 towards Patients with Type II Rickets Harboring VDR (R274L) cDNA

As described in the previous sections, AH-1 showed a high binding ability to VDR (R274L) and a high resistance to CYP24A1-dependent metabolism. These results suggest that AH-1 could demonstrate therapeutic effects on type II rickets caused by VDR (R274L). Currently, we administered AH-1 to VDR (R270L) rats, and the expected results have been obtained (data not shown).

### 4.6. Elucidation of Molecular Mechanism Vitamin D Actions by Comparison among the GM Rats

**Figure 6:**
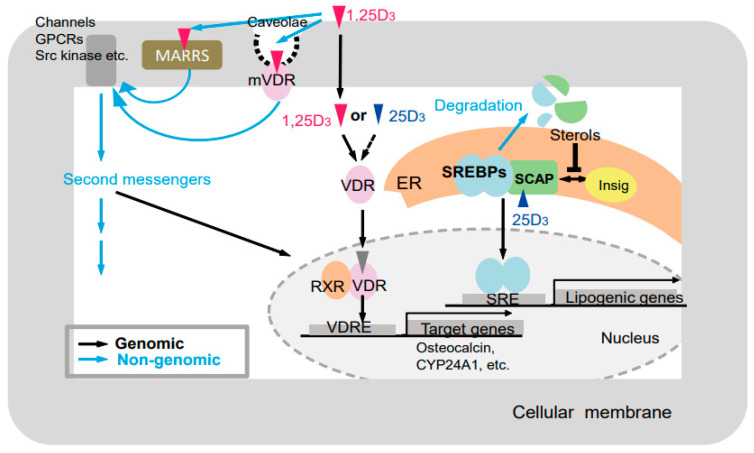
Putative modes of action of vitamin D [25]. Black and blue arrows indicate genomic and nongenomic pathways, respectively. GPCRs, G-protein-coupled receptor; MARRS, (membrane-associated, rapid response steroid-binding) receptor; VDR, vitamin D receptor; mVDR, membrane-bound vitamin D receptor; RXR, retinoid X receptor; VDRE, vitamin D response element; ER, endoplasmic reticulum; SREBPs, sterol regulatory-element–binding proteins; SCAP, SREBP cleavage-activating protein; SRE, sterol regulatory element.

Various vitamin D actions could be elucidated by comparing physiological conditions, such as bone and skin formation, and multiple serum parameters, such as Ca, P, 25(OH)D_3_, and PTH, in the GM rats generated in this study (Figure 6). Previous studies have showed genomic and nongenomic actions of vitamin D mediated by VDR [78,79], and VDR-independent actions of vitamin D [80]. The VDR-independent effect of 25(OH)D_3_ on lipid metabolism by inducing degradation of SREBP/SCAP was recently reported. In addition, ligand-independent effects of VDR have been reported [81]. Thus, at least five types of effects of vitamin D and/or the VDR should be considered, namely: (1) VDR-dependent effects of 1α,25(OH)_2_D_3_ [19,20], (2) VDR-independent effects of 1α,25(OH)_2_D_3_ [80], (3) VDR-dependent effects of 25(OH)D_3_ (VDR-25(OH)D_3_) [82], (4) VDR-independent effects of 25(OH)D_3_ [83], and (5) ligand-independent effects of VDR (Table 1) [81].

Comparison between wild-type and *Vdr* (R270L) rats could reveal (1) VDR-dependent 1α,25(OH)_2_D_3_ effects (Table 1). Comparison between *Vdr* (R270L) and *Cyp27b1*-KO rats may reveal (2) VDR-independent effects of 1α,25(OH)_2_D_3_. In addition, comparison between *Vdr* (R270L) and *Vdr-*KO rats may reveal (3) VDR-dependent effects of 25(OH)D_3_ or (5) ligand-independent effects of the VDR. Thus, our GM rats appear to be useful for the elucidation of molecular mechanism vitamin D actions and the development of vitamin D derivatives for clinical treatment.

## 5. Conclusions

The vitamin D derivative evaluation systems we have developed in this study are quite useful. They can readily measure VDR affinity and CYP24A1-mediated metabolism. In addition, the GM rats we have generated by genome editing are highly useful for evaluating the efficacy, safety, and pharmacokinetics of vitamin D derivatives. The reasons rats were used in this study instead of mice include their much larger body size and greater blood volume relative to mice, rendering rats more suitable for pharmacokinetic studies. We hope these evaluation systems will contribute to the near-future development of drugs with excellent therapeutic potential.

## Figures and Tables

**Table 1:** Vitamin D and/or VDR actions observed in WT and GM rats [25].

Rat Strain	Mode of Action of Vitamin D				
	(1)	(2)	(3)	(4)	(5)
	Vdr-1,25D3	non Vdr-1,25D3	Vdr-25D3	non Vdr-25D3	Vdr-no ligand
WT	+	+	+	+	+
*Vdr* (R270L)	−	+	+	+	+
*Cyp27b1*-KO	−	−	+	+	+
*Vdr*-KO	−	+	−	+	−

Vdr-1,25D3; Vdr-dependent action of: non-Vdr-1,25D3; Vdr-independent action of: Vdr-25D3; Vdr-dependent action of 25(OH)D3: non-Vdr-25D3; Vdr-independent action of 25(OH)D3: Vdr-no ligand; ligand-independent action of Vdr.
